# Supplementary material for: TTDCapsNet: Tri Texton-Dense Capsule Network for complex and medical image recognition
Source: PLoS One. 2024 Mar 15;19(3):e0300133. doi: 10.1371/journal.pone.0300133 (PMC10942055; doi:10.1371/journal.pone.0300133)
Supplement: Attachment — Submitted filename: Response to Reviewers.docx [file pone.0300133.s001.docx]

**Original Manuscript ID:** PONE-D-23-06111

**Original Article Title: “**TTDCapsNet: Tri Texton-Dense Capsule Network for Complex image recognition”

**To:** PLOS ONE

**Re:** Response to reviewers

Dear Editor,

We would like to express our profound gratitude to the Editor and the reviewers for their professional and constructive comments on our manuscript, “TTDCapsNet: Tri Texton-Dense Capsule Network for Complex image Recognition” *(ID:* PONE-D-23-06111). We have revised the manuscript according to the comments and suggestions of the associate Editor and the reviewers, and have responded to the reviewer’s concerns. The responses to the reviewer’s comments are therefore attached in separate sheets.

Thank you for allowing a resubmission of our manuscript, with an opportunity to address the reviewers’ comments.

We are uploading (a) our point-by-point response to the comments (below) (response to reviewers), (b) an updated manuscript with yellow and green highlighting indicating changes for made as suggested by Reviewer1 and 2 respectively (some of the changes to be made overlap), and (c) a clean, updated manuscript without highlights (PDF main document). Finally, the authors would like to affirm your office that the manuscript can be assigned to the same reviewers. For further information, please feel free to contact the corresponding author. Her personal information is as follows:

**Mailing address:**	Ms. Vivian Akoto-Adjepong

Department of Computer Science and Informatics,

University of Energy and Natural Resources,

P.O. Box 214, Sunyani - Ghana.

**Phone number:**	+233 (0) 247385738

**Email:**	vivian.akoto-adjepong@uenr.edu.gh

Best regards

Vivian Akoto-Adjepong et al.

Reviewer #1: 1. Points in favor

i. The article contributes to knowledge and may be considered for publication after carrying out the following suggestions to improve the paper

**Response:** Thank you for the review

ii. Overall, the article is ok

**Response:** Thank you for the review

**Reviewer 1, Comment # 1:** The abstract needs to be rewritten in one paragraph stating clearly what is done, how its done and results/limitations

**Response:** Thank you for the review. In the revised manuscript, the abstract has been rewritten in one paragraph stating clearly what is done, how it’s done, and the results. **[see page: 1]**

**Author action:**

Convolutional Neural Networks (CNNs) are frequently used algorithms because of their propensity to learn relevant and hierarchical features through their feature extraction technique. However, the availability of enormous volumes of data in various variations is crucial for their performance. Capsule networks (CapsNets) perform well on a small amount of data but perform poorly on complex images. To address this, we proposed a new Capsule Network architecture called Tri Texton-Dense CapsNet (TTDCapsNet) for better complex image classification. The TTDCapsNet is made up of three hierarchic blocks of Texton-Dense CapsNet (TDCapsNet) models. A single TDCapsNet is a CapsNet architecture composed of a texton detection layer to extract essential features, which are passed onto an eight-layered block of dense convolution that further extracts features, and then the output feature map is given as input to a Primary Capsule (PC), and then to a Class Capsule (CC) layer for classification. The resulting feature map from the first PC serves as input into the second-level TDCapsNet, and that from the second PC serves as input into the third-level TDCapsNet. The routing algorithm receives feature maps from each PC for the various CCs. Routing the concatenation of the three PCs creates an additional CC layer. All these four feature maps combined, help to achieve better classification. On fashion-MNIST, CIFAR-10, Breast Cancer, and Brain Tumor datasets, the proposed model is evaluated and achieved validation accuracies of 94.90\%, 89.09\%, 95.01\%, and 97.71\% respectively. Findings from this work indicate that TTDCapsNet outperforms the baseline and performs comparatively well with the state-of-the-art CapsNet models using different performance metrics. This work clarifies the viability of using Capsule Network on complex tasks in the real world. Thus, the proposed model can be used as an intelligent system, to help oncologists in diagnosing cancerous diseases and administering treatment required.

**Reviewer 1, Comment # 2:** The introduction is not properly written. A good introduction should lead the reader from a generalized topic to a particular aspect. It helps to establish the main idea, context, and research importance and summarizes background data on the topic, providing the primary goal of the work. Also, the contribution of the paper is not clearly stated.

**Response:** Thank you for the review. In the revised manuscript, the introduction has been written properly to improve the readability of the work, and the contribution of the paper is also clearly stated. Find some portions of the introduction below. **[see page:2- 3]**

**Author action:**

Deep learning models [10, 11] have been applied to many domains including medical health, to aid specialists, and make the classification of such diseases (malignant) much easier. They must increase performance on complicated images in terms of convergence, accuracy, flexibility, robustness, and complexity before it can be helpful to oncologists in carrying out the procedure of malignant illness identification effectively. CNNs are well-known and frequently used deep learning models because of their propensity to learn relevant and hierarchical features through their feature extraction technique with convolution structures [12]. They have been applied in the medical field, in diagnosing diseases such as Acne and Rosacea [13–15], brain tumors [16–18], breast cancer [19–21], etc. **[see page: 2]**

The idea of ” capsules” was first proposed to address the issues of CNN [23, 25] **[see page: 2]**

Therefore, there is a need to improve the existing capsule network algorithm to help solve the problem of crowding, in order to classify complex images well or with precision. This paper utilizes the dynamic routing algorithm of Capsule Network by incorporating a texton layer [30] to extract essential features, which are passed onto an eight (8)-layered block of dense convolution that further extracts features to improve the texture, color, and spatial recognition capabilities of Capsule Network, by enabling the decision of crucial features, and the needed coupling coefficients to be decreased in order to improve the hierarchical relationship among capsules that are related closely for better classification of complex images such as medical images. Fig 1 depicts the workflow adopted for the proposed work, which depicts the testing and training processes. **[see page: 2-3]**

This paper’s main contributions are as follows:

1) An improved, flexible, and robust Capsule Network which incorporates texton and eight (8)-layered blocks of dense convolution for effective feature extraction is proposed.

2) Excellent color, edge, and texture extraction algorithms texton and dense convolution were suggested for better feature extraction, to help in identifying affected parts of medical images using Capsule Network.

3) A comparative analysis was conducted to evaluate the proposed model with other deep learning models, using metrics such as specificity, sensitivity, precision, accuracy, and others. **[see page: 3]**

**Reviewer 1, Comment # 3**: The literature review needs to be improved, and the presentation style should be flow of thought. Consider the following to improve the body of literature

1. DOI: 10.1371/journal.pone.0282812

2. DOI: 10.1007/s42235-022-00316-8

3. DOI: 10.33003/fjs-2022-0603-990

4. DOI: 10.1007/s11831-022-09850-4

5. DOI: 10.1371/journal.pone.0275346

6. DOI: 10.1007/s00521-022-07854-6

**Response:** Thank you for the review. In the revised manuscript, the authors have improved the literature review and the presentation style. **[see page: 3] [see page:5] [see page: 8]**

**Author action:**

This review starts with capsule networks proposed for the recognition of open datasets for deep learning models, CIFAR-10, Fashion MNIST, etc., and other datasets. **[see page: 3]**

Other authors proposed CapsNet and other deep-learning models in order to identify malignant conditions for breast cancer and brain tumor detection. **[see page: 5]**

All the proposed models performed well on the various datasets. But for medical image diagnosis, there is a need for a more robust and efficient model for better diagnosis, hence this study aims to propose an improved, flexible, and robust Capsule Network which incorporates texton and eight (8)-layered blocks of dense convolution for effective feature extraction, for better classification of malignant diseases. **[see page: 8]**

**Reviewer 1, Comment # 4:** All Figures need to be explained in more detail as it relates to the study.

**Response:** Thank you for the review. In the revised manuscript, all figures have been explained in detail as it relates to the study. Here are some of the explanations. **[see page: 12, 14]**

**Author action:**

The proposed model’s performance is assessed with that of the traditional CapsNet [28]. The accuracy and loss graphs for the proposed and baseline CapsNet models are displayed in Fig 7. It is clear from the accuracy graphs that the proposed model outperforms the baseline CapsNet model in terms of accuracy and early convergence. This means that the proposed model predicts correctly more than the baseline model. In comparison to the proposed model validation accuracy, which is greater and consistent, the baseline validation accuracy for breast cancer experienced lower accuracy with significant swings. Also, contrary to the proposed model, the baseline CapsNet model for CIFAR-10 exhibits a steady fall in test accuracy following convergence.

Fig 8 displays the ROC and area under the curve graphs for fashion-MNIST, CIFAR-10, Breast Cancer, and Brain Tumor. The proposed model outperformed the baseline model when the values for the ROC and Precision-Recall curves were taken into account. This shows that, the proposed model is more robust.

The confusion matrices, which are depicted in Fig 9, indicate how many images were correctly and incorrectly identified. We calculated the true positive, false positive, true negative, false negative, accuracy per class values, specificity, sensitivity, and precision to extract relevant information from these figures. In the proposed model, fewer images were incorrectly identified than in the baseline CapsNet model, as shown in Fig 9. It is clear from the true positive and accuracy numbers for each class that the suggested model performed well across all of the classes.

For the accuracy per class using the breast cancer dataset, for both classes 0 and 1 using the baseline model, 28.89% of the images belonging to these classes were wrongly classified as compared to the proposed model that only misclassified 4.99% of the images belonging to these classes. For the brain tumor dataset, misclassification for classes 0, 1, 2, and 3 using the baseline model was 3.43%, 4.04%, 0.76%, and 0.3% respectively, whiles the proposed model misclassifications were 1.45%, 1.75%, 0.91%, and 0.45% respectively for the same classes. This shows that the proposed model classifies images belonging to the various classes better than the baseline model. Also, for precision using the breast cancer dataset, for both classes 0 and 1 using the baseline model, 27.59% and 28.96% of images predicted to belong to classes 0 and 1 respectively do not belong to those classes, compared to the proposed model, where only 8.71% and 3.2% images for classes 0 and 1 respectively does not belong to those classes. For the brain tumor, using the baseline model, 5.57%, 9.58%, 1.99%, and 0.98% of images for classes 0, 1, 2, and 3 respectively, did not actually belong to these classes compared to 2.05%, 2.99%, 2.94%, and 1.46% of images for classes 0, 1, 2, and 3 respectively, when using the proposed model. It can be seen that the proposed model had fewer images predicted that do not belong to their respective classes than the baseline model. Again, for sensitivity using the breast cancer dataset, for both classes 0 and 1 with the baseline model, 87.3% and 2.21% images belonging to these classes are missed, compared to 7.06% and 4.05% of images missed for classes 0 and 1 respectively using the proposed model. For the brain tumor dataset, using the baseline model, 9.67%, 7.52%, 1.33%, and 0% of images for classes 0, 1, 2, and 3 respectively are missed as compared to 4.33%, 4.57%, 1%, and 0% of images for classes 0, 1, 2, and 3 respectively, missed using the proposed model. This shows that, the proposed model did not miss many images belonging to the various classes, but correctly identified images belonging to particular classes as opposed to the baseline model. Also, for specificity using the breast cancer dataset, for both classes 0 and 1 with the baseline model, 2.21% and 87.3% of images were misclassified as belonging to the various classes respectively, whiles the proposed model misclassified 4.05% and 7.06% images for the various classes respectively as belonging to those classes, whiles they do not belong to them. For the brain tumor, using the baseline model, 1.58%, 2.98%, 0.59%, and 0.44% of images for classes 0,1,2,3 respectively, were misclassified as belonging to the various classes, compared to 0.59%, 0.89%, 0.89%, and 0.66% images for classes 0,1,2,3 respectively, that were misclassified to belong to the various classes by the proposed model whiles they did not belong to them. These figures obtained, show that the proposed model was able to exclude images that do not belong to a particular class better than the baseline model. All this can be found in Table 1 and Table 2., whose values were retrieved from confusion matrices found in Fig 9. Furthermore, the proposed model gives better ROC values than the baseline model, which indicates that the proposed model is capable of distinguishing between classes at various thresholds. Also, the precision-recall values for the proposed model were better, compared to that of the baseline model as shown in Fig 8. These graphs help to access a model’s robustness, when the dataset is imbalanced as can be found in the breast cancer dataset. Overall, the proposed model showed very promising results in terms of accuracy, reliability, and likelihood of false positive and negative predictions, compared to the baseline model, which is a very needed property, in medical image diagnosis. These promising results can be attributed to the good feature extraction capabilities (texton and dense layers) that were employed to extract only relevant information from the various images subjected to the model.

**Reviewer 1, Comment # 5:** The result and discussion section are poorly written. The authors need to explain or link the finding with their proposed contributions and how it differs from what is in the available literature.

**Response:** Thank you for the review. In the revised manuscript, the result and discussion section has been rewritten well to link the finding with the proposed contribution and how it differs from what is available in the literature. **[see page: 15]**

**Author action:**

As shown in Table 4, considering the fashion-MNIST dataset, the proposed model that employed texton and dense layers for better feature extraction obtained the highest validation accuracy of 94.90%, followed by Abra and colleagues’ proposed CapsNet model [45] that used Gabor and preprocessing blocks for feature extraction and the least validation accuracy of 90.26% was obtained from Ozan and colleagues proposed model [43] that used quaternions to design a CapsNet model. For CIFAR-10 dataset, a CapsNet model by Phaye and colleagues [41], that used dense blocks for feature extraction achieved the highest validation accuracy of 89.71, followed by the proposed model that achieved a validation accuracy of 89.09%, and the least validation accuracy is from the baseline CapsNet by Sabour and colleagues [28], which uses a convolution layer for feature extraction. For the breast cancer dataset, the proposed model had the highest validation accuracy of 95.01% as compared to 71.11% of the basline model. Lastly, for the Brain tumor dataset, the proposed model had the highest validation accuracy of 97.71% followed by the baseline model at 95.73%, and then the model proposed by Phaye and colleagues obtained the least validation accuracy of 95.03% [41]. The proposed model performs commensurately well compared to the various state-of-the-art capsule network models. The proposed model outperformed the baseline model by 4.18%, 26.18% 23.9%, and 1.98% for fashion-MNIST, CIFAR10, Breast Cancer, and Brain Tumor datasets. This desirable performance required for complex image (e.g., medical image) diagnosis by the proposed model is attributed to the good feature extraction capabilities (texton and dense layers) that were employed to extract only relevant information from the various images subjected to the model.

**Reviewer 1, Comment # 6:** There may be a need to find the statistical significance of the claimed improvement of this work

**Response:** Thank you for the review. In the revised manuscript, the statistical significance of the improvement made has been stated clearly. **[see page: 12, 14]**

**Author action:**

The proposed model’s performance is assessed with that of the traditional CapsNet [28]. The accuracy and loss graphs for the proposed and baseline CapsNet models are displayed in Fig 7. It is clear from the accuracy graphs that the proposed model outperforms the baseline CapsNet model in terms of accuracy and early convergence. This means that the proposed model predicts correctly more than the baseline model. In comparison to the proposed model validation accuracy, which is greater and consistent, the baseline validation accuracy for breast cancer experienced lower accuracy with significant swings. Also, contrary to the proposed model, the baseline CapsNet model for CIFAR-10 exhibits a steady fall in test accuracy following convergence.

Fig 8 displays the ROC and area under the curve graphs for fashion-MNIST, CIFAR-10, Breast Cancer, and Brain Tumor. The proposed model outperformed the baseline model when the values for the ROC and Precision-Recall curves were taken into account. This shows that, the proposed model is more robust.

The confusion matrices, which are depicted in Fig 9, indicate how many images were correctly and incorrectly identified. We calculated the true positive, false positive, true negative, false negative, accuracy per class values, specificity, sensitivity, and precision to extract relevant information from these figures. In the proposed model, fewer images were incorrectly identified than in the baseline CapsNet model, as shown in Fig 9. It is clear from the true positive and accuracy numbers for each class that the suggested model performed well across all of the classes.

For the accuracy per class using the breast cancer dataset, for both classes 0 and 1 using the baseline model, 28.89% of the images belonging to these classes were wrongly classified as compared to the proposed model that only misclassified 4.99% of the images belonging to these classes. For the brain tumor dataset, misclassification for classes 0, 1, 2, and 3 using the baseline model was 3.43%, 4.04%, 0.76%, and 0.3% respectively, whiles the proposed model misclassifications were 1.45%, 1.75%, 0.91%, and 0.45% respectively for the same classes. This shows that the proposed model classifies images belonging to the various classes better than the baseline model. Also, for precision using the breast cancer dataset, for both classes 0 and 1 using the baseline model, 27.59% and 28.96% of images predicted to belong to classes 0 and 1 respectively do not belong to those classes, compared to the proposed model, where only 8.71% and 3.2% images for classes 0 and 1 respectively does not belong to those classes. For the brain tumor, using the baseline model, 5.57%, 9.58%, 1.99%, and 0.98% of images for classes 0, 1, 2, and 3 respectively, did not actually belong to these classes compared to 2.05%, 2.99%, 2.94%, and 1.46% of images for classes 0, 1, 2, and 3 respectively, when using the proposed model. It can be seen that the proposed model had fewer images predicted that do not belong to their respective classes than the baseline model. Again, for sensitivity using the breast cancer dataset, for both classes 0 and 1 with the baseline model, 87.3% and 2.21% images belonging to these classes are missed, compared to 7.06% and 4.05% of images missed for classes 0 and 1 respectively using the proposed model. For the brain tumor dataset, using the baseline model, 9.67%, 7.52%, 1.33%, and 0% of images for classes 0, 1, 2, and 3 respectively are missed as compared to 4.33%, 4.57%, 1%, and 0% of images for classes 0, 1, 2, and 3 respectively, missed using the proposed model. This shows that, the proposed model did not miss many images belonging to the various classes, but correctly identified images belonging to particular classes as opposed to the baseline model. Also, for specificity using the breast cancer dataset, for both classes 0 and 1 with the baseline model, 2.21% and 87.3% of images were misclassified as belonging to the various classes respectively, whiles the proposed model misclassified 4.05% and 7.06% images for the various classes respectively as belonging to those classes, whiles they do not belong to them. For the brain tumor, using the baseline model, 1.58%, 2.98%, 0.59%, and 0.44% of images for classes 0,1,2,3 respectively, were misclassified as belonging to the various classes, compared to 0.59%, 0.89%, 0.89%, and 0.66% images for classes 0,1,2,3 respectively, that were misclassified to belong to the various classes by the proposed model whiles they did not belong to them. These figures obtained, show that the proposed model was able to exclude images that do not belong to a particular class better than the baseline model. All this can be found in Table 1 and Table 2., whose values were retrieved from confusion matrices found in Fig 9. Furthermore, the proposed model gives better ROC values than the baseline model, which indicates that the proposed model is capable of distinguishing between classes at various thresholds. Also, the precision-recall values for the proposed model were better, compared to that of the baseline model as shown in Fig 8. These graphs help to access a model’s robustness, when the dataset is imbalanced as can be found in the breast cancer dataset. Overall, the proposed model showed very promising results in terms of accuracy, reliability, and likelihood of false positive and negative predictions, compared to the baseline model, which is a very needed property, in medical image diagnosis. These promising results can be attributed to the good feature extraction capabilities (texton and dense layers) that were employed to extract only relevant information from the various images subjected to the model.

**Reviewer 1, Comment # 5:** The result and discussion section are poorly written. The authors need to explain or link the finding with their proposed contributions and how it differs from what is in the available literature.

**Response:** Thank you for the review. In the revised manuscript, the result and discussion section has been rewritten well to link the finding with the proposed contribution and how it differs from what is available in the literature. **[see page: 15]**

**Author action:**

As shown in Table 4, considering the fashion-MNIST dataset, the proposed model that employed texton and dense layers for better feature extraction obtained the highest validation accuracy of 94.90%, followed by Abra and colleagues’ proposed CapsNet model [45] that used Gabor and preprocessing blocks for feature extraction and the least validation accuracy of 90.26% was obtained from Ozan and colleagues proposed model [43] that used quaternions to design a CapsNet model. For CIFAR-10 dataset, a CapsNet model by Phaye and colleagues [41], that used dense blocks for feature extraction achieved the highest validation accuracy of 89.71, followed by the proposed model that achieved a validation accuracy of 89.09%, and the least validation accuracy is from the baseline CapsNet by Sabour and colleagues [28], which uses a convolution layer for feature extraction. For the breast cancer dataset, the proposed model had the highest validation accuracy of 95.01% as compared to 71.11% of the basline model. Lastly, for the Brain tumor dataset, the proposed model had the highest validation accuracy of 97.71% followed by the baseline model at 95.73%, and then the model proposed by Phaye and colleagues obtained the least validation accuracy of 95.03% [41]. The proposed model performs commensurately well compared to the various state-of-the-art capsule network models. The proposed model outperformed the baseline model by 4.18%, 26.18% 23.9%, and 1.98% for fashion-MNIST, CIFAR10, Breast Cancer, and Brain Tumor datasets. This desirable performance required for complex image (e.g., medical image) diagnosis by the proposed model is attributed to the good feature extraction capabilities (texton and dense layers) that were employed to extract only relevant information from the various images subjected to the model.

**Reviewer 1, Comment # 7:** The conclusion may need to be improved, relating it to the contributions and findings. Also, state some limitations

**Response:** Thank you for the review. In the revised manuscript, the conclusion has been improved, relating it to the contributions and findings. Also, some limitations have been stated. **[see page: 16]**

**Author action:**

An improved Capsule Network model with a texton detection method and dense layers was proposed in this paper to help extract key features and increase the recognition rate on complex images. The adoption of texton detection method and dense layers helped to improve the model’s feature extraction abilities, thus helping to prevent overfitting due to class imbalance, acquiring competitive validation accuracies, and obtaining performances that are appreciable. The proposed model outperformed the CapsNet baseline model and performed comparatively well with the state-of-the-art models in literature on the four datasets in terms of convergence, accuracy, flexibility, robustness, and complexity. When tested against the fashion-MNIST, CIFAR-10, breast cancer, and brain tumor datasets, the suggested model performed admirably. In general, the results of this study clarify the viability of using Capsule Networks on complex tasks in the real world. However, the routing algorithm used by the capsule network has been blamed for decreasing its expressivity as well as hindering it from input distinguishing, which limits the effective classification of the model. In the future, a new routing algorithm coupled with fewer model parameters will be considered to improve the routing of vectors from lower-level to higher-level capsules for better classification and feasible implementation on smartphones.

**Reviewer #2:** - Spell out each acronym the first time used in the Abstract (CNN) as well as the body of the paper

**Response:** Thank you for the review. In the revised manuscript, each acronym that was not spelled out the first time has been spelled out **[see page: 1] [see page: 2]**

**Author action:**

Convolutional Neural Networks (CNNs) **[see page: 1]**

**Magnetic Resonance Imaging (MRI)** **[see page: 2]**

**Reviewer 2, Comment # 1**: The author should discuss more recent references in the introduction as below.

*Gaze Prediction Based on Convolutional Neural Network

*Radiologists versus Deep Convolutional Neural Networks: A Comparative Study for Diagnosing COVID-19

*Accurate and compact convolutional neural network based on stochastic computing

*Automated Diagnosis of Acne and Rosacea using Convolution Neural Networks

*Deterministic Modeling to Predict the Natural Gas Density Using Artificial Neural Networks

**Response:** Thank you for the review. In the revised manuscript, the introduction has been improved based on the recommended paper titled “Automated Diagnosis of Acne and Rosacea using Convolution Neural Networks”. Although the rest of the recommended papers are good, they do not directly relate to the focus and the application area of the study. To improve the introduction of this study authors have also discussed other recent works related to the study. **[see page: 2]**

**Author action:**

CNNs are well-known and frequently used deep learning models because of their propensity to learn relevant and hierarchical features through their feature extraction technique with convolution structures [12]. They have been applied in the medical field, in diagnosing diseases such as Acne and Rosacea [13–15], brain tumors [16–18], breast cancer [19–21], etc.

**Reviewer 2, Comment # 2:** The author should improve figures 1 and 2. background of all images should be white.

**Response:** Thank you for the review. In the revised manuscript figure 1 and 2 has been improved and the background of all images has been made white. **[see page: 8]**

**Author action:**

Figure 1 **[see page: 8]**

Figure 2 **[see page: 8]**

**Reviewer 2, Comment # 3:** Authors should remove title above the Figures 7 and 8. if the same as the caption otherwise it can be written as the caption. and also, background

**Response:** Thank you for the review. In the revised manuscript, the titles of Figures 7 and 8 have been removed. **[see page: 14]**

**Author action:**

Figure 7: **[see page: 14]**

Figure 8: **[see page: 14]**

**Reviewer 2, Comment # 4:** graphs of figure 7 and 8 are not looking good. author should improve the quality.

**Response:** Thank you for the review. In the revised manuscript the quality of Figures 7 and 8 have been improved by the authors. **[see page: 14]**

**Author action:**

Figure 7: **[see page: 14]**

Figure 8: **[see page: 14]**

**Reviewer 2, Comment # 5:** Conclusion to be made more systematic, and future scope to be elaborated more on technical features that are planned to be added in the proposed system in the near future.

**Response: Response:** Thank you for the review. In the revised manuscript, the conclusion has been improved to be more systematic, and the future scope has been elaborated. Figure 8: **[see page: 16]**

**Author action:**

An improved Capsule Network model with a texton detection method and dense layers was proposed in this paper to help extract key features and increase the recognition rate on complex images. The adoption of texton detection method and dense layers helped to improve the model’s feature extraction abilities, thus helping to prevent overfitting due to class imbalance, acquiring competitive validation accuracies, and obtaining performances that are appreciable. The proposed model outperformed the CapsNet baseline model and performed comparatively well with the state-of-the-art models in literature on the four datasets in terms of convergence, accuracy, flexibility, robustness, and complexity. When tested against the fashion-MNIST, CIFAR-10, breast cancer, and brain tumor datasets, the suggested model performed admirably. In general, the results of this study clarify the viability of using Capsule Networks on complex tasks in the real world. However, the routing algorithm used by the capsule network has been blamed for decreasing its expressivity as well as hindering it from input distinguishing, which limits the effective classification of the model. In the future, a new routing algorithm coupled with fewer model parameters will be considered to improve the routing of vectors from lower-level to higher-level capsules for better classification and feasible implementation on smartphones.

**Reviewer 2, Comment # 6:** In the Conclusion, future directions and challenges should be explained more.

**Response: Thank you for the review. In the revised manuscript, future directions and challenges are explained more in the conclusion [see pages: 16]**

**Author action:**

An improved Capsule Network model with a texton detection method and dense layers was proposed in this paper to help extract key features and increase the recognition rate on complex images. The adoption of texton detection method and dense layers helped to improve the model’s feature extraction abilities, thus helping to prevent overfitting due to class imbalance, acquiring competitive validation accuracies, and obtaining performances that are appreciable. The proposed model outperformed the CapsNet baseline model and performed comparatively well with the state-of-the-art models in literature on the four datasets in terms of convergence, accuracy, flexibility, robustness, and complexity. When tested against the fashion-MNIST, CIFAR-10, breast cancer, and brain tumor datasets, the suggested model performed admirably. In general, the results of this study clarify the viability of using Capsule Networks on complex tasks in the real world. However, the routing algorithm used by the capsule network has been blamed for decreasing its expressivity as well as hindering it from input distinguishing, which limits the effective classification of the model. In the future, a new routing algorithm coupled with fewer model parameters will be considered to improve the routing of vectors from lower-level to higher-level capsules for better classification and feasible implementation on smartphones.

**Reviewer 2, Comment # 7:** please give a proofread check to the paper.

**Response:** Thank you for the review. In the revised manuscript, thorough proofreading has been done by the authors.
