# Supplementary material for: TTDCapsNet: Tri Texton-Dense Capsule Network for complex and medical image recognition
Source: PLoS One. 2024 Mar 15;19(3):e0300133. doi: 10.1371/journal.pone.0300133 (PMC10942055; doi:10.1371/journal.pone.0300133)
Supplement: Attachment — Submitted filename: Response to Reviewers.docx [file pone.0300133.s002.docx]

**Original Manuscript ID:** PONE-D-23-06111

**New Manuscript ID: PONE-D-23-06111R1**

**Original Article Title: “**TTDCapsNet: Tri Texton-Dense Capsule Network for Complex Image Recognition”

**To:** PLOS ONE

**Re:** Response to reviewers

Dear Editor,

We would like to express our profound gratitude to the Editor and the reviewers for their professional and constructive comments on our manuscript, “TTDCapsNet: Tri Texton-Dense Capsule Network for Complex image Recognition” *(ID:* PONE-D-23-06111R1). We have revised the manuscript according to the comments and suggestions of the associate Editor and the reviewers, and have responded to the reviewer’s concerns. The responses to the reviewer’s comments are therefore attached in separate sheets.

Thank you for allowing a resubmission of our manuscript, with an opportunity to address the reviewers’ comments.

We are uploading (a) our point-by-point response to the comments (below) (response to reviewers), (b) an updated manuscript with yellow, blue, and green highlighting indicating changes made as suggested by Reviewers 3,4, and 5 respectively, (some of the comments to be addressed overlap) and (c) a clean, updated manuscript without highlights (PDF main document). Finally, the authors would like to affirm your office that the manuscript can be assigned to the same reviewers. For further information, please feel free to contact the corresponding author. Her personal information is as follows:

**Mailing address:**	Ms. Vivian Akoto-Adjepong

Department of Computer Science and Informatics,

University of Energy and Natural Resources,

P.O. Box 214, Sunyani - Ghana.

**Phone number:**	+233 (0) 247385738

**Email:**	vivian.akoto-adjepong@uenr.edu.gh

Best regards

Vivian Akoto-Adjepong et al.

**Reviewer #3**: From the experimental data, it can be seen that the proposed model lacks effective innovation. The authors can conduct comparative experiments with models from recent years to demonstrate the advantages of the proposed model.

**Response:** Thank you for the review. In the revised manuscript, a comparative analysis with models from recent years (2020, 2021, 2022, 2023) has been done **[see page: 17]**

**Author action:**

| Methods | Validation accuracy (%) | | | |
| --- | --- | --- | --- | --- |
|  | fashion-MNIST | CIFAR 10 | Breast Cancer | Brain Tumor |
| ShallowNet (Mensah et al., 2021) | 92.70 | 75.75 |  |  |
| CapsNet (Sabour et al., 2017) | 90.72 | 62.91 | 71.11 | 95.73 |
| Enhanced-CapsNet (Zhang et al., 2020) | - | 82.31 | - | - |
| 64 Capsule Layers (Xi et al., 2017) | - | 64.67 | - | - |
| Feature Amplification CapsNet (Abra et al., 2021) | 93.76 | 84.56 | - | - |
| Multi-lane Chang & Liu (2020) | 92.63 | 76.79 | - | - |
| MS-CapsNet (Xiang et al., 2018) | 92.70 | 75.70 | - | - |
| ResCapsNet (Goswami and Sun, 2019) | - | 78.54 | - | - |
| CFC-CapsNet (Shiri and Baniasadi (2021) | 92.86 | 73.15 | - | - |
| Fast Inference (Zhao et al., 2019) | 91.52 | 70.33 | - | - |
| Inverted dot product (Tsai et al., 2020) | - | 82.55 | - | - |
| DCNET++ (Phaye et al., 2018) | 94.65 | 89.71 | - | 95.03 |
| Max–min (Zhao et al. (2019a) | 92.07 | 75.92 | - | - |
| Quaternion CapsNet (Ozan et al., 2020) | 90.26 | 82.21 | - | - |
| Gabor capsule network with preprocessing blocks (Abra Ayidzoe et al., 2021) | 94.78 | 85.24 | - | - |
| SqueezeCapsNet (Adu et al, 2023) | 93.49 | 82.45 |  |  |
| Afriyie et al 2022a | 92.8 | 75.42 |  |  |
| Afriyie et al 2022b | 94.93 | 84.57 |  |  |
| MCNet (Qinghai et al 2023) | 93.17 | 79.27 |  |  |
| **Proposed Model** | **94.90** | **89.09** | **95.01** | **97.71** |

**[see page: 17]**

**Reviewer #4**: The authors proposed a CapsNet-based technique suitable for complex image classification. The abstract and introduction are well-written. Moreover, the contribution of the study is clearly indicated. The literature survey is comprehensive. The methodology is well-written, and the proposed technique is properly explained. The authors can improve the paper as follows:

**Reviewer 4, Comment # 1:** One of the key advantages of CapsNet over CNN is its robustness to image transformations. To further demonstrate the robustness of the proposed technique, the authors can compare the proposed model with state-of-the-art CNN-based architecture such as DenseNet, ResNet, etc. It will be interesting to see which of the algorithms are more robust to image transformation.

**Response:** Thank you for the review. In the revised manuscript a comparison of the proposed model to state-of-the-art CNN models have been done to prove the robustness of the proposed model **[see page: 17-18]**

**Author action:**

**4.5 Transformed data learning and transformation robustness**

The purpose and impact of transformations applied to the training and testing datasets serve distinct objectives. Transformations during testing can serve as a means to assess a model's robustness to transformations. Conversely, transformations during training serve as a way to evaluate a model's capacity to learn from these transformations. Both aspects contribute to understanding a model's transformation robustness and the learning ability from data that is transformed. In this research, we introduced random transformations to the train set of the fashion-MNIST and CIFAR-10 datasets, including translations of up to 2 pixels in both the horizontal and vertical directions and random rotations within the (-180°, 180°) range. This is denoted as a tuple (2, 180°). To assess model robustness, we created a test set with translational shifts of 2 pixels and rotational variations within 90°, represented as a tuple (2, 90°). To ensure a comprehensive evaluation of the proposed model's robustness, we included two CNN models, ResNet-18-aug and ResNet-34-aug, which were trained with augmentation techniques, alongside the baseline CapsNet model. The performance of the two CNN baseline models on fashion-MNIST and CIFAR-10 datasets has been documented in (Venkataraman et al., 2023), and these results serve as points of reference for comparison in Table [5].

**Table 5:** Comparison of validation accuracies in % for transformation robustness of CapsNet and CNN based models

|  | **fashion-MNIST** | | **CIFAR-10** | |
| --- | --- | --- | --- | --- |
| **Model** | **(0, 0)** | **(2, 90°)** | **(0, 0)** | **(2, 90°)** |
| ResNet-18-aug | 94.21 | 93.30 | 78.84 | 79.60 |
| ResNet-34-aug | 94.38 | 93.78 | 81.27 | 81.60 |
| CapsNet (Sabour et al., 2017) | 90.72 | 88.52 | 62.91 | 61.46 |
| Proposed Model | 94.90 | 94.74 | 89.09 | 88.86 |

From Table 5, it can be seen that, even though there are small reductions of 0.16% and 0.23% in the validation accuracies 94.90% and 89.09% obtained for fashion-MNIST and CIFAR-10 transformed image datasets respectively for the proposed model. The proposed model generalized very well on the transformed unseen data, hence achieving the highest validation accuracies on the two datasets. This shows the proposed models’ robustness to image transformation. Also, the three baseline models also performed comparatively well in terms of robustness to image transformation. There was a reduction in the validation accuracies 94.21% and 94.38% by 0.91% and 0.60% respectively, when using fashion-MNIST transformed image dataset for the two CNN baseline models ResNet-18-aug and ResNet-34-aug respectively, but an increase in the validation accuracies 78.84% and 81.27% by 0.76% and 0.33 % respectively when using the same CNN baseline models on CIFAR-10 transformed image dataset. Again, the baseline CapsNet model when used, recorded a reduction in validation accuracy by 1.45% and 2.2% when fashion-MNIST and CIFAR-10 transformed image datasets were run with the model respectively. Comparing the proposed model to the baseline CNN and CapsNet models, it can clearly be seen that, the proposed model is more robust to image transformation. This splendid performance can be attributed to the equivariant nature of CapsNet, as well as the effective feature extraction capabilities of the proposed model. **[see page: 17-18]**

**Reviewer 4, Comment # 2:** The authors reported the complexity of the proposed technique. They should also compare the complexity of the proposed technique to that of baseline CapsNet. Which is more optimal? Which is more time-consuming?

**Response:** Thank you for the review. In the revised manuscript, the complexity of the proposed and baseline model has been compared in terms of optimality and time-consumption **[see page: 16]**

**Author action:**

The number of parameters generated for the various datasets in millions for the proposed model are 11.89, 14.53, 8.88, 10.29, and that for the baseline model are 8.22, 11.75, 4.93, and 9.43 for fashion-MNIST, CIFAR-10, Breast Cancer and Brain Tumor datasets respectively. It can be seen that; the proposed model generates more parameters and hence spends more time in training than the baseline model, but the proposed model is more optimal in terms of precision, sensitivity, specificity, accuracy, ROC, and PR curve values. **[see page: 16]**

**Reviewer 4, Comment # 3:** Why did the authors scale the images in the brain cancer dataset to 32x33x3? What was the original image size? The scaling will affect the image quality and subsequently affect the performance of the model.

**Response:** Thank you for the review. The authors resized the input image from 512 x 512 x3 to 32 x 32 x 3. This is because resizing images is a critical pre-processing step in computer vision. Principally, deep learning models train faster on small images. A larger input image requires the neural network to learn from four times as many pixels, and this increase the training time and computational complexity of the architecture (Saponara and Elhanashi, 2022).

Reference

Saponara, S., Elhanashi, A. (2022). Impact of Image Resizing on Deep Learning Detectors for Training Time and Model Performance. In: Saponara, S., De Gloria, A. (eds) Applications in Electronics Pervading Industry, Environment and Society. ApplePies 2021. Lecture Notes in Electrical Engineering, vol 866. Springer, Cham. <https://doi.org/10.1007/978-3-030-95498-7_2>

**Reviewer 4, Comment # 4:** The authors presented a comparative performance analysis between the proposed technique and baseline CapsNet. Before comparing the two algorithms, the authors should also provide a detailed performance analysis of the proposed technique. They should discuss the performance using all performance metrics presented in the paper. The authors should report and clearly explain the results.

**Response:** Thank you for the review. In the revised manuscript the detailed performance analysis of the proposed technique is done using all the performance metrics presented in the paper. **[see page: 13]**

**Author action:**

From figure 7, the proposed model performed very well in terms of accuracy, since it obtained high validation accuracies of 94.90%, 89.09%, 95.01%, and 97.71% for fashion-MNIST, CIFAR-10, Breast Cancer, and Brain Tumor datasets, representing the correct predictions from the entire predictions made. This shows that, the proposed model generalizes very well on unseen data. Furthermore, confusion matrices of the proposed model are depicted in Figure 9. This indicates how many images were correctly and incorrectly identified. The diagonal cells highlighted in blue represent accurate predictions made by the model, including both true positives and true negatives. Misclassifications produced by the model are shown in white, both above and below the correct predictions. We calculated the true positive, false positive, true negative, false negative, accuracy per class values, specificity, sensitivity, and precision to extract relevant information from these figures. Considering figure 9, Table 1, and Table 2, it can be seen that the proposed model classifies images belonging to the various classes for the Brain Tumor and Breast Cancer datasets better, with high validation accuracies per class. It can be seen that; the proposed model had less images predicted that does not belong to their respective classes, hence the high values of precisions attained by the various classes in the datasets when using the proposed model. Also, the proposed model did not miss much images belonging to the various classes, but correctly identified images belonging to particular classes, hence the higher values obtained for sensitivity or recall for the various classes in the datasets. Again, the proposed model was able to exclude images that does not belong to a particular class in the datasets. This is indicated by the high specificity values obtained by the various classes in the dataset when the proposed model is used. Moreover, from Figure 8 the Area under the curve (AUC) graphs for fashion-MNIST, CIFAR 10 are shown. The proposed model shows better ROC values for all the classes in the datasets, which indicates that the proposed model is capable of distinguishing between classes at various threshold, also, the precision recall values (that help to access the performance of a model on imbalanced dataset as can be found in the Breast Cancer dataset) for the proposed model were high for all the datasets. This shows that, proposed model is robust and performs well on imbalanced dataset as can be found in the breast cancer dataset. In general, the proposed model demonstrated highly encouraging outcomes in accuracy, reliability, and likelihood of false positive and negative predictions, which are very needed characteristics in medical image diagnosis. **[see page: 13]**

**Reviewer 4, Comment # 5:** The authors should compare the proposed technique and the baseline CapsNet based on their robustness to image transformation, overfitting, generalization performance, etc.

**Response:** Thank you for the review. In the revised manuscript, the proposed and baseline CapsNets are compared in terms of image transformation, overfitting, generalization performance etc. **[see page: 17-18]**

**Author action:**

**4.5 Transformed data learning and transformation robustness**

The purpose and impact of transformations applied to the training and testing datasets serve distinct objectives. Transformations during testing can serve as a means to assess a model's robustness to transformations. Conversely, transformations during training serve as a way to evaluate a model's capacity to learn from these transformations. Both aspects contribute to understanding a model's transformation robustness and the learning ability from data that is transformed. In this research, we introduced random transformations to the train set of the fashion-MNIST and CIFAR-10 datasets, including translations of up to 2 pixels in both the horizontal and vertical directions and random rotations within the (-180°, 180°) range. This is denoted as a tuple (2, 180°). To assess model robustness, we created a test set with translational shifts of 2 pixels and rotational variations within 90°, represented as a tuple (2, 90°). To ensure a comprehensive evaluation of the proposed model's robustness, we included two CNN models, ResNet-18-aug and ResNet-34-aug, which were trained with augmentation techniques, alongside the baseline CapsNet model. The performance of the two CNN baseline models on fashion-MNIST and CIFAR-10 datasets has been documented in (), and these results serve as points of reference for comparison in Table [5].

Table 5: Comparison of validation accuracies in % for transformation robustness of CapsNet and CNN based models

|  | **fashion-MNIST** | | **CIFAR-10** | |
| --- | --- | --- | --- | --- |
| **Model** | **(0, 0)** | **(2, 90°)** | **(0, 0)** | **(2, 90°)** |
| ResNet-18-aug | 94.21 | 93.30 | 78.84 | 79.60 |
| ResNet-34-aug | 94.38 | 93.78 | 81.27 | 81.60 |
| CapsNet (Sabour et al., 2017) | 90.72 | 88.52 | 62.91 | 61.46 |
| Proposed Model | 94.90 | 94.74 | 89.09 | 88.86 |

From Table 5, it can be seen that, even though there are small reductions of 0.16% and 0.23% in the validation accuracies 94.90% and 89.09% obtained for fashion-MNIST and CIFAR-10 transformed image datasets respectively for the proposed model. The proposed model generalized very well on the transformed unseen data, hence achieving the highest validation accuracies on the two datasets. This shows the proposed models’ robustness to image transformation. Also, the three baseline models also performed comparatively well in terms of robustness to image transformation. There was a reduction in the validation accuracies 94.21% and 94.38% by 0.91% and 0.60% respectively, when using fashion-MNIST transformed image dataset for the two CNN baseline models ResNet-18-aug and ResNet-34-aug respectively, but an increase in the validation accuracies 78.84% and 81.27% by 0.76% and 0.33 % respectively when using the same CNN baseline models on CIFAR-10 transformed image dataset. Again, the baseline CapsNet model when used, recorded a reduction in validation accuracy by 1.45% and 2.2% when fashion-MNIST and CIFAR-10 transformed image datasets were run with the model respectively. Comparing the proposed model to the baseline CNN and CapsNet models, it can clearly be seen that, the proposed model is more robust to image transformation. This splendid performance can be attributed to the equivariant nature of CapsNet, as well as the effective feature extraction capabilities of the proposed model. **[see page: 17-18]**

It can be seen from figure 7 that, the validation accuracies of the proposed model are higher for all the datasets than the baseline model. This means that, the proposed model obtained higher correct predictions from the entire number of predictions, hence, generalizes well on unseen data than the baseline model. Considering figure 7e and 7f, representing accuracy and loss for Breast Cancer dataset, it can be seen that, the baseline model overfitted and experienced lower validation accuracy with significant swings in contrast to the proposed model’s graphs that experienced early convergence and higher accuracy. This means that, the baseline model excelled at fitting the training data for Breast Cancer dataset, but struggled to generalize to new or unseen Breast Cancer data during validation. **[see page: 14]**

**Reviewer 4, Comment # 6:** If I am interpreting Figure 7a correctly, there seems to be a substantial difference between the training and validation accuracy. If I am correct, the proposed model will not generalize well on unseen images. Kindly provide a discussion of the generalization performance of the proposed model. **[see page: 14]**

**Response:** Thank you for the review. Validation accuracy is the accuracy of a model on new data, while training accuracy is the accuracy of a model on the data it was trained on. Typically, validation accuracy is lower than training accuracy, as the model has never seen the validation data before. The dataset was split into two. 80% for training and 20% for validation. From Figure 7a, the proposed models training accuracy (red) and the validation accuracy (green) whiles the traditional CapsNet training accuracy (blue) and validation accuracy (violet). The proposed model can generalize well on unseen data than the traditional or baseline model since the validation accuracy (accuracy on unseen data) is better than that of the baseline letter.

**Author action:**

It can be seen from figure 7 that, the validation accuracies of the proposed model are higher for all the datasets than the baseline model. This means that, the proposed model obtained higher correct predictions from the entire number of predictions, hence, generalizes well on unseen data than the baseline model. Considering figure 7e and 7f, representing accuracy and loss for Breast Cancer dataset, it can be seen that, the baseline model overfitted and experienced lower validation accuracy with significant swings in contrast to the proposed model’s graphs that experience early convergence and higher accuracy. This means that, the baseline model excelled at fitting the training data for Breast Cancer dataset, but struggled to generalize to new or unseen Breast Cancer data during validation. **[see page: 14]**

**Reviewer 5, Comment # 1:** This manuscript is a detailed and rigorous revision of the previously mentioned comment.

**Response:** Thank you for the review.

**Reviewer 5, Comment # 2:** In the "Proposed model" section, the reference to "Fig 5. Architecture of the proposed CapsNet model." is not shown in the paper, so please check it and add it in time.

**Response:** Thank you for the review. In the revised manuscript, Fig 5 has been referenced. **[see page: 10]**

**Author action:** The proposed Tri Texton-Dense CapsNet (TTDCapsNet) model (shown in Fig 5)
consists of three Texton detection layers, three eight-layered dense convolutional blocks,
three primary capsule layers, four class capsule layers, and a decoder network.

**Reviewer 5, Comment # 3:** In the "Performance evaluation "section, the confusion matrix presented in Fig 9, shows the number of correctly and incorrectly categorized images. In order to better understand the results of the confusion matrix, it is recommended to provide an explanation of each element in the confusion matrix.

**Response:** Thank you for the review. In the revised manuscript, from performance evaluation measures section, Confusion Matrix (CM) section, metrics such as True Positive (TP), True Negative (TN), False Positive (FP), False Negative (FN), accuracy, precision, sensitivity, and specificity, that can be obtained from the confusion matrices are explained. Also, the explanation of the elements in the confusion matrices used to obtain these metrics mentioned above are made.

**Author action:**

True positive (TP): Correct prediction with positive actual value.

False positive (FP): Wrong prediction with positive actual value

True negative (TN): Correct prediction with negative actual value

False negative (FN): Wrong prediction with negative actual value **[see page: 12]**

The diagonal cells highlighted in blue represent accurate predictions made by the models, including both true positives and true negatives. Misclassifications produced by the model are shown in white, both above and below the correct predictions. **[see page: 13]**

**Reviewer 5, Comment # 4:** In the Capsule network section of Proposed methods, there is a lack of detailed explanations for many specialized knowledge, such as the principles of the routing algorithms, how the coupling coefficients are calculated, the protocols between the capsules, and the details of how to update the coupling coefficients.

**Response:** Thank you for the review. In the revised manuscript detailed explanations of principles of the routing algorithm, how the coupling coefficients are calculated, the protocols between the capsules and how the coupling coefficients are updated has been done. **[see page: 8 -9]**

**Author action:**

**Capsule Network**

A capsule (Sabour et al., 2017) is a collection of neurons, where the activity vector length denotes the probability of an entity existing, and the direction of the vector denotes the instantiation parameters. A CNN layer comes first in a capsule network, followed by a Primary Capsule (PC) layer, then Classification is handled by the Class Capsule (C) layer and Reconstruction is handled by the decoder network which is fully connected. The CNN layer does the feature extraction and the output is given as input into the primary capsule layer. Each capsule $i$ (where*:* $1\leq i\leq N$ ) present in the *l* layer has activity vector $u_{i}\epsilon\mathbb{R}$, which in instantiation parameter form, encodes the spatial information. The $i^{th}$ lower-level capsule’s output vector $u_{i}$, is given as input to all upper layer capsules in the$l + 1$ layer. $u_{i}$ will be received by layer $l + 1$s’ $j^{th}$ capsule, and the product $W_{ij}$ will be found. The product of activity vector $u_{i}$ and its weight matrix $W_{ij}$ is the primary capsule output $\hat{u}_{j|i}=W_{ij}u_{i}$. With the coupling coefficient $C_{ij}=\frac{\mathrm{ex}p \left( b_{ij} \right)}{\sum_{k} \mathrm{ex}p \left( b_{ik} \right)}$, which is the SoftMax of the logits $b_{ij}=b_{ij}+v_{j} .\hat{u}_{j|i}$, the primary capsule predictions are connected to a suitable class capsule. Upon agreement $a_{ij}=v_{j} .\hat{u}_{j|i}$among a lower-level and a higher-level prediction, $C_{ij}$ get updated in the course of the dynamic routing process indicated in Algorithm 1. A higher-level capsule $j$entire input is the weighed-sum of every vector predicted $\hat{u}_{j|i}$of a specific primary capsule $i$ for a specific class capsule $j$, this is given by $s_{j}=\sum_{i=1}^{N} c_{ij} .\hat{u}_{j|i}$, which represents the vector input to the $j^{th}$ capsule. A non-linear squashing function $v_{j}=\frac{{{||s}_{j}||}^{2}}{{{1+||s}_{j}||}^{2}}\frac{s_{j}}{{||s}_{j}||}$ is used to limit the output value of the Class Capsule between [0,1], in order to prevent vector outputs from exceeding 1. Hence $v_{j}$ represents the output vector showing the probability of the vector between 0 and 1. In the last layer, a capsule labeled $k$ is connected with a loss $l_{k}$

The final layer contains Capsule $k$, which is associated with a loss denoted as lk. This leads to a significant loss when capsules exhibit extended output instantiation parameters in cases where the entity being represented does not actually exist. The expression for the loss function $l_{k}$ is found in the equation below;

$$L_{k}=T_{k} {max(0,m}^{+}-{{||v}_{k}||)}^{2}+\lambda\left( {1-T}_{k} \right) max\left( {0, ||v}_{k}||-m^{-} \right)^{2}$$

Where *T_k_*=1 when class *k* is active and 0 otherwise. Hyperparameters *λ*, *m^-^*, *m^+^* are set during the process of learning. On a variety of problems, CapsNets shown in Figure 2, have shown good performance (Kwabena Patrick et al., 2022).

**Algorithm 1:** Dynamic Routing-by-Agreement

1. $\mathbf{Procedure} \mathrm{ROUTING} (\hat{u}_{j|i}\boldsymbol{,}r, l)$
2. $\mathbf{For} all capsule i in layer l and capsule j in layer (l+ 1) : b_{ij} \leftarrow0$
3. $\mathbf{For} r \mathrm{iterations} \boldsymbol{do}$
4. $\mathbf{For} all capsule i in layer l :c_{i}\leftarrow SoftMax\left( b_{i} \right) ⊳softmax computes c_{ij}$
5. $\mathbf{For} all capsule j in layer (l+ 1) :s_{j}\leftarrow\sum_{i=1}^{N} c_{ij} .\hat{u}_{j|i}$
6. $\mathbf{For} all capsule j in layer (l+ 1) :v_{j}\leftarrow squash\left( s_{j} \right)⊳squash computes v_{j}$
7. $\mathbf{For}all capsule i in layer l and capsule j in layer \left( l+1 \right):b_{ij}=b_{ij}+v_{j}.\hat{u}_{j|i}$
8. $\boldsymbol{return}v_{j}$

**Reviewer 5, Comment # 5:** In the experimental section, comparative experiments and ablation experiments are conducted, and It is rigorous and scientific in explaining the results and evaluation indexes in detail.

**Response:** Thank you for the review.
