# Supplementary material for: TTDCapsNet: Tri Texton-Dense Capsule Network for complex and medical image recognition
Source: PLoS One. 2024 Mar 15;19(3):e0300133. doi: 10.1371/journal.pone.0300133 (PMC10942055; doi:10.1371/journal.pone.0300133)
Supplement: Attachment — Submitted filename: Response to Reviewers.docx [file pone.0300133.s003.docx]

**Original Manuscript ID:** PONE-D-23-06111

**3^rd^ Revision**

**New Manuscript ID:** PONE-D-23-06111R2

**Original Article Title: “**TTDCapsNet: Tri Texton-Dense Capsule Network for Complex Image Recognition”

**To:** PLOS ONE

**Re:** Response to reviewers

Dear Editor,

We would like to express our profound gratitude to the Editor and the reviewers for their professional and constructive comments on our manuscript, “TTDCapsNet: Tri Texton-Dense Capsule Network for Complex image Recognition” *(ID:* PONE-D-23-06111R2). We have revised the manuscript according to the comments and suggestions of the associate Editor and the reviewers, and have responded to the reviewer’s concerns. The responses to the reviewer’s comments are therefore attached in separate sheets.

Thank you for allowing a resubmission of our manuscript, with an opportunity to address the reviewers’ comments.

We are uploading (a) our point-by-point response to the comments (below) (response to reviewers), (b) an updated manuscript with green highlighting indicating changes made as suggested by Reviewers 5, and (c) a clean, updated manuscript without highlights (PDF main document). Finally, the authors would like to affirm your office that the manuscript can be assigned to the same reviewers. For further information, please feel free to contact the corresponding author. Her personal information is as follows:

**Mailing address:**	Ms. Vivian Akoto-Adjepong

Department of Computer Science and Informatics,

University of Energy and Natural Resources,

P.O. Box 214, Sunyani - Ghana.

**Phone number:**	+233 (0) 247385738

**Email:**	vivian.akoto-adjepong@uenr.edu.gh

Best regards

Vivian Akoto-Adjepong et al.

**Reviewer #5: Comment 1**: This manuscript has been comprehensively revised and improved based on previously proposed suggestions.

**Response:** Thank you for the review.

**Reviewer #5: Comment 2:** To make the literature study comprehensive, the authors may refer to more similar research in the final publication version, for example, “Multi-lane capsule network for classifying images with complex background” “A novel dense capsule network based on dense capsule layers”.

**Response:** Thank you for the review. In the revised manuscript, the literature is made comprehensive by referring to more similar research and then used for comparative analysis. **[see pages: 5 and 18]**

**Author action:**

Chang and team innovatively crafted a capsule-oriented network architecture, introducing modifications to the Squash function and refining the dropout implementation. Thorough experimentation was undertaken on three widely-used datasets—MNIST, affNIST, and CIFAR10—yielding accuracies of 99.73%, 81.71%, and 76.79%, respectively [98]. Also, Sun and colleagues introduced an innovative dense capsule network called DenseCaps, which utilizes dense capsule layers. The architecture comprises three Dense Capsule Blocks to facilitate the reuse of features and multi-scale feature capsule processing calculation of loss. The model demonstrated notable performance with an accuracy of 94.93% on fashion-MNIST and 89.41% on CIFAR-10 [99]. Through the incorporation of the Convolutional Capsule Layer (Conv-Caps Layer), Xiong and team enhanced CapsNet, leading to a significant boost in its overall performance. This enhancement resulted in validation accuracies of 81.29% for CIFAR-10 and 99.84% for MNIST datasets [100]. Do and colleagues presented a multi-lane capsule network (MLCN) designed for parallel processing, delivering notable accuracy with reduced costs. The MLCN comprises distinct parallel lanes, each contributing to a specific result dimension. Evaluation of the model on fashion-MNIST and CIFAR-10 datasets demonstrated validation accuracies of 92.63% and 75.18%, respectively [101]. CHENG and colleagues introduced the Complex-valued Diverse CapsNet (Cv-CapsNet++), which involves a three-stage process. Initially, a restricted dense complex-valued subnetwork is utilized to acquire multi-scale complex-valued features. Subsequently, these features are encoded into complex-valued primary capsules in the second stage. Lastly, in the third stage, they extended the dynamic routing algorithm to the complex-valued domain, employing it to integrate real- and imaginary-valued information from the complex-valued primary capsules. The model achieved validation accuracies of 94.40% on Fashion-MNIST and 86.70% on CIFAR-10 datasets [102]. Shiri and her team substituted the second convolutional layer with a fully connected (FC) layer and directed the result of the initial convolutional layer into another FC layer. The FC layer's output was subsequently transformed to generate Primary Capsules, serving as inputs for the dynamic routing algorithm. Notably, the model attained validation accuracies of 88.84% and 67.18% on the fashion-MNIST and CIFAR-10 datasets, respectively [103]. Huang and Zhou introduced a capsule network with a dual attention mechanism, referred to as DA-CapsNet. In this model, the initial attention layer follows the convolution layer, while the subsequent attention layer is incorporated after the primary capsule layer. The model demonstrated notable validation accuracies of 93.98% and 85.47% on the fashion-MNIST and CIFAR-10 datasets respectively [104]. Luo and his team introduced R-CapsNet, drawing inspiration from the impact of a compact convolution kernel. This design serves as an expansion of the initial CapsNet, featuring four convolutional layers and one fully connected layer. During testing on the fashion-MNIST and CIFAR-10 datasets, the model demonstrated validation accuracies of 93.89% and 81.2% respectively [105].	**[see page: 5]**

| Methods | Validation accuracy (%) | | | |
| --- | --- | --- | --- | --- |
|  | fashion-MNIST | CIFAR 10 | Breast Cancer | Brain Tumor |
| ShallowNet [33] | 92.70 | 75.75 |  |  |
| CapsNet [28] | 90.72 | 62.91 | 71.11 | 95.73 |
| Enhanced-CapsNet [34] | - | 82.31 | - | - |
| 64 Capsule Layers [31] | - | 64.67 | - | - |
| Feature Amplification CapsNet [35] | 93.76 | 84.56 | - | - |
| Multi-lane [32] | 92.63 | 76.79 | - | - |
| MS-CapsNet [36] | 92.70 | 75.70 | - | - |
| ResCapsNet [37] | - | 78.54 | - | - |
| CFC-CapsNet [38] | 92.86 | 73.15 | - | - |
| Fast Inference [39] | 91.52 | 70.33 | - | - |
| Inverted dot product [40] | - | 82.55 | - | - |
| DCNET++ [41] | 94.65 | 89.71 | - | 95.03 |
| Max–min [42] | 92.07 | 75.92 | - | - |
| Quaternion CapsNet [43] | 90.26 | 82.21 | - | - |
| Gabor capsule network with prep blocks [45] | 94.78 | 85.24 | - | - |
| SqueezeCapsNet [93] | 93.49 | 82.45 | - | - |
| Afriyie et al [94] | 92.8 | 75.42 | - | - |
| MCNet [95] | 93.17 | 79.27 | - | - |
| Afriyie et al [96] | 94.93 | 84.57 | - | - |
| MLSCN [98] | - | 76.79 | - | - |
| DenseCaps [99] | 94.93 | 89.41 | - | - |
| DeeperCaps [100] | - | 81.29 | - | - |
| MLCN [101] | 92.63 | 75.18 | - | - |
| Cv-CapsNet++ [102] | 94.40 | 86.70 | - | - |
| Quick-CapsNet (QCN) [103] | 88.84 | 67.18 | - | - |
| DA-CapsNet [104] | 93.98 | 85.47 | - | - |
| R-CapsNet [105] | 93.89 | 81.57 | - | - |
| **Proposed Model** | **94.90** | **89.09** | **95.01** | **97.71** |

**[see page: 18]**

**Reviewer #6:** The manuscript proposed a Capsule Network architecture called Tri Texton-Dense CapsNet (TTDCapsNet) for better complex image classification. The manuscript is carefully written and has addressed all issues raised.

**Response:** Thank you for the review.

**Reviewer #7**: (No Response)

**Response:** Thank you for the review.
