# Supplementary material for: TTDCapsNet: Tri Texton-Dense Capsule Network for complex and medical image recognition
Source: PLoS One. 2024 Mar 15;19(3):e0300133. doi: 10.1371/journal.pone.0300133 (PMC10942055; doi:10.1371/journal.pone.0300133)
Supplement: Attachment — Submitted filename: Response to Reviewers.docx [file pone.0300133.s004.docx]

**Original Manuscript ID:** PONE-D-23-06111

**Manuscript ID:** PONE-D-23-06111R3

**Original Article Title: “**TTDCapsNet: Tri Texton-Dense Capsule Network for Complex and Medical Image Recognition”

**To:** PLOS ONE

**Re:** Response to reviewers

Dear Editor,

We would like to express our profound gratitude to the Editor and the reviewers for their constructive comments on our manuscript, “TTDCapsNet: Tri Texton-Dense Capsule Network for Complex and Medical Image Recognition” *(ID:* PONE-D-23-06111R2). We have revised the manuscript according to the comments and suggestions of the associate Editor, and have responded to the concerns. Thank you for allowing a resubmission of our manuscript.

We are uploading (a) our response to the comments (below) (response to reviewers), (b) an updated manuscript with *green* highlighting indicating changes, and (c) a clean, updated manuscript without highlights (PDF manuscript). For further information, please feel free to contact the corresponding author. Her personal information is as follows:

**Mailing address:**	Ms. Vivian Akoto-Adjepong

Department of Computer Science and Informatics,

University of Energy and Natural Resources,

P.O. Box 214, Sunyani - Ghana.

**Phone number:**	+233 (0) 247385738

**Email:**	vivian.akoto-adjepong@uenr.edu.gh

Best regards

Vivian Akoto-Adjepong et al.

**Editor Comment**: We note that you do not appear to have responded to the previously stated PLOS ONE requirements regarding code sharing.

**Response:** Thank you for the review. In the revised manuscript, all author-generated code that underpins the findings in the manuscript has been publicly made available without restrictions. Also, datasets used in this study have been provided. This can be found under Data Availability **[see page: 20]**

**Author action:**

Guidelines for code sharing were followed, to help ensure that, the code that underpins the findings in the manuscript is shared in a way that follows best practice and facilitates reproducibility and reuse.

*The code can be found in the link below;*

<https://github.com/vivianakotoadjepong/TTDCapsNet.git>

Links to datasets used in this study;

Fashion-MNIST; <https://www.kaggle.com/datasets/zalando-research/fashionmnist>

CIFAR-10; <https://www.cs.toronto.edu/~kriz/cifar.html>

Breast Cancer; <https://www.kaggle.com/datasets/ambarish/breakhis>

Brain Tumor; <https://www.kaggle.com/datasets/masoudnickparvar/brain-tumor-mri-dataset>
